# Murine Typhus Masquerading as Thrombotic Thrombocytopenic Purpura in a Patient With Newly Diagnosed HIV

**DOI:** 10.7759/cureus.116679

**Published:** 2026-09-21

**Authors:** Jibreel Abdul Cader, Shannon M Lee, Arnold Tsai

**Affiliations:** 1 Medicine, David Geffen School of Medicine - UCLA, Los Angeles, USA; 2 Medicine, Charles R. Drew University of Medicine and Science, Los Angeles, USA; 3 Internal Medicine, Harbor UCLA, Torrance, USA

**Keywords:** acquired immune deficiency syndrome (aids), hiv, murine typhus, rickettsial disease, rickettsia typhi

## Abstract

Murine typhus is a flea-borne rickettsial disease caused by *Rickettsia typhi*. It presents with nonspecific symptoms and laboratory derangements, which can lead to a delay in diagnosis and treatment. We report the case of a 58-year-old man who presented with fever, altered mental status, nausea, vomiting, and diarrhea. Laboratory testing revealed thrombocytopenia, acute renal failure requiring emergent hemodialysis, transaminitis, and hyperbilirubinemia. An intermediate PLASMIC score initially prompted a concern for thrombotic thrombocytopenic purpura, and urgent plasma exchange was initiated after the first round of dialysis, but this was ruled out when ADAMTS13 (a disintegrin and metalloproteinase with a thrombospondin type 1 motif, member 13) activity was low. During hospitalization, he was newly diagnosed with HIV, with a CD4 count of 65 cells/mm^3^. Given his fever, he was started on empiric doxycycline for presumed rickettsial infection in the setting of his symptoms, immunocompromised state, and lab abnormalities while confirmatory testing was pending. He completed a 10-day course of doxycycline, was started on antiretroviral therapy, and was discharged after recovery of renal function and resolution of thrombocytopenia, transaminitis, and changes in mental status. This case demonstrates that severe murine typhus can cause multi-organ failure in patients with newly diagnosed HIV/AIDS. Prompt empirical doxycycline, initiated before serologic confirmation, is essential to prevent progression to critical illness.

## Introduction

Murine typhus is an infection caused by *Rickettsia typhi*, which is transmitted to humans via the bite of infected fleas, with rats and opossums serving as amplifying reservoirs rather than vectors [1]. It exhibits a distinct epidemiological pattern, commonly affecting urban, port, and coastal regions, including California [1]. Although historically regarded as uncommon, murine typhus is a reemerging disease in the United States, with cases concentrated in warm coastal areas of California and Texas [2]. Reported cases in Los Angeles County have risen steadily since 2010, reaching 171 in 2022, the year of the most recent local fatality cluster [3]. Its true incidence is likely underestimated because the presentation is nonspecific and flea exposure is frequently unrecognized by patients [4].

Rickettsial infection targets vascular endothelial cells, leading to microvascular injury, tissue hypoperfusion, and microthrombus formation [1,5,6]. In immunocompetent hosts, murine typhus classically presents with a triad of fever, headache, and maculopapular rash that begins on the trunk and spreads to the extremities, sparing the palms and soles [7,8]. However, this triad is present in only about one-third of patients, and the diagnosis more often rests on a recognizable but nonspecific laboratory signature: a tetrad of elevated hepatic transaminases, elevated lactate dehydrogenase, elevated erythrocyte sedimentation rate, and hypoalbuminemia, frequently accompanied by thrombocytopenia and hyponatremia [4,7]. This constellation overlaps substantially with sepsis and with the thrombocytopenia and organ injury of a thrombotic microangiopathy.

This presentation can be difficult to distinguish from other causes of thrombocytopenia and multi-organ dysfunction, including thrombotic thrombocytopenic purpura (TTP). Thrombocytopenia with microangiopathic organ injury defines the thrombotic microangiopathies (TMAs), a group that extends well beyond TTP to include Shiga-toxin-mediated and complement-mediated (atypical) hemolytic uremic syndrome, disseminated intravascular coagulation (DIC), drug-induced TMA, malignancy, pregnancy-associated TMA, and autoimmune disease [9,10]. Systemic infection is a particularly important and under-recognized TTP mimic: a systematic review of infection-associated TTP/HUS-like presentations identified 41 distinct infectious etiologies, including cytomegalovirus, HIV, ehrlichiosis, and Rocky Mountain spotted fever (RMSF), organisms that, like *R. typhi*, injure the vascular endothelium [11]. Other infectious causes of an acute febrile, thrombocytopenic, multi-organ illness relevant to this setting include leptospirosis, dengue, and other rickettsioses [7].

Data on murine typhus in people with HIV are limited. A serosurvey of human immunodeficiency virus (HIV)-infected patients found *R. typhi* seroprevalence and clinical correlates comparable to the general population, with no association with CD4 count or CDC stage [12]. Large clinical series characterizing severe murine typhus have generally comprised immunocompetent patients or explicitly excluded untreated HIV infection, and, thus, whether advanced HIV/acquired immunodeficiency syndrome (AIDS) independently worsens severity, produces atypical presentations, or increases complications has not been established [4,13].

This case describes a severe manifestation of murine typhus that closely mimicked TTP in a patient with newly diagnosed AIDS. In this patient, the pivot from TTP to murine typhus rested on two steps. First, TTP was effectively excluded: severe (<10%) ADAMTS13 (a disintegrin and metalloproteinase with a thrombospondin type 1 motif, member 13) deficiency is specific for TTP, whereas the mild-to-moderate reductions seen in sepsis, DIC, liver disease, and other secondary TMAs are not [14,15]; our patient's ADAMTS13 activity was low but not severely deficient (0.51 IU/mL), making TTP unlikely and prompting discontinuation of plasma exchange. Second, once a primary TMA was excluded, the combination of fever, transaminitis, thrombocytopenia, acute kidney injury, and newly recognized advanced HIV in a resident of an endemic coastal region redirected attention to a rickettsial and opportunistic-infection differential, prompting empiric doxycycline and confirmatory *R. typhi* serology.

This case was previously presented as a poster at the Solomon Scholars Internal Medicine Conference at the University of California, Los Angeles on May 18, 2026.

## Case presentation

A 58-year-old man with no prior medical history presented with generalized weakness, poor oral intake, nausea, vomiting, and non-bloody diarrhea. He worked as a butcher and landscaper, lived in Los Angeles, California, and reported engaging in sexual activity with men. He denied recent travel, known allergies, prescribed or over-the-counter medications, or substance use. On presentation, he was febrile (38.3°C), tachycardic (heart rate of 101 beats per minute), and tachypneic (respiratory rate of 20 breaths per minute), and had a blood pressure of 128/69 mmHg. Physical examination revealed a man groaning and wincing with scattered petechiae over the bilateral lower extremities and altered mental status with intermittent confusion on admission.

Initial laboratory testing revealed leukocytosis, thrombocytopenia, elevated creatinine levels with hyperkalemia, and transaminitis with hyperbilirubinemia. The HIV-1 antibody test result was also positive. Electrocardiography revealed a first-degree atrioventricular block and peaked T waves (Figure 1). Computed tomography (CT) of the head was unremarkable; CT of the chest revealed a 7-mm nodule versus nodular opacity in the left upper lobe (Figure 2); CT of the abdomen and pelvis showed mild hepatomegaly (Figure 3). Subsequent right upper quadrant ultrasound revealed hepatic steatosis (Figure 4). Blood, urine, and stool cultures were obtained, and empiric vancomycin, ceftriaxone, and metronidazole were initiated for presumed sepsis of gastrointestinal origin

**Figure 1:**
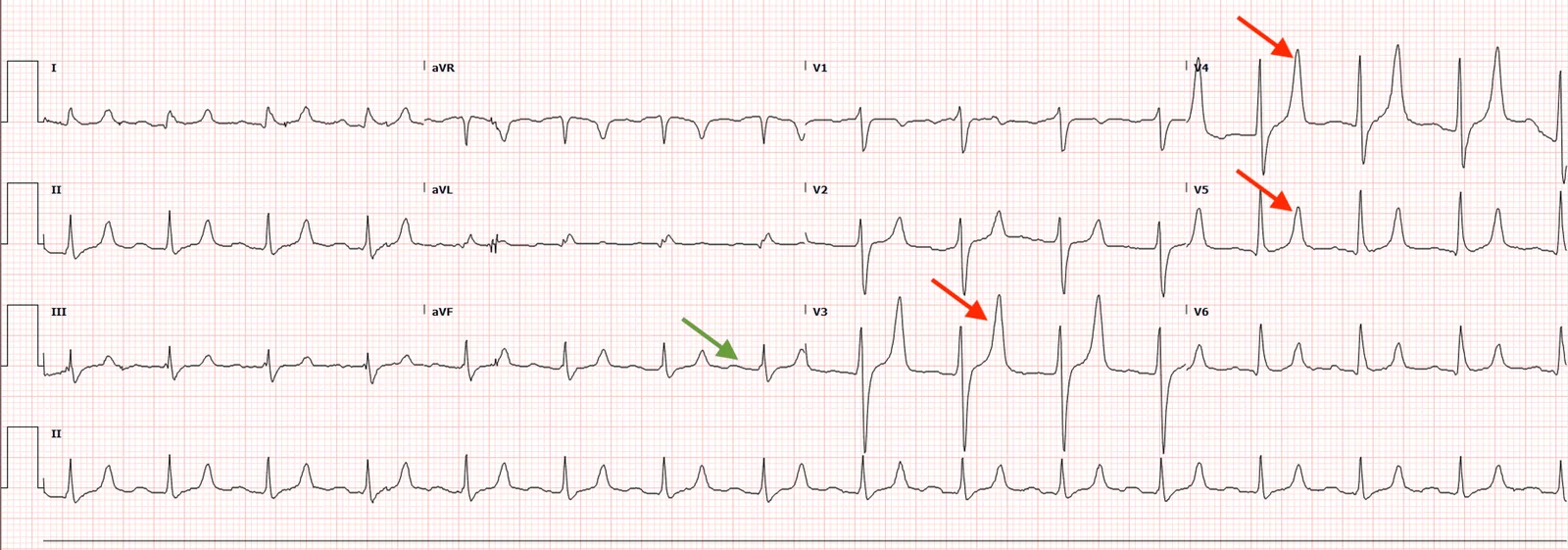
Electrocardiography at admission Normal sinus rhythm with first-degree atrioventricular block (green arrow) and peaked t-waves (red arrows), findings consistent with this patient's hyperkalemia.

**Figure 2:**
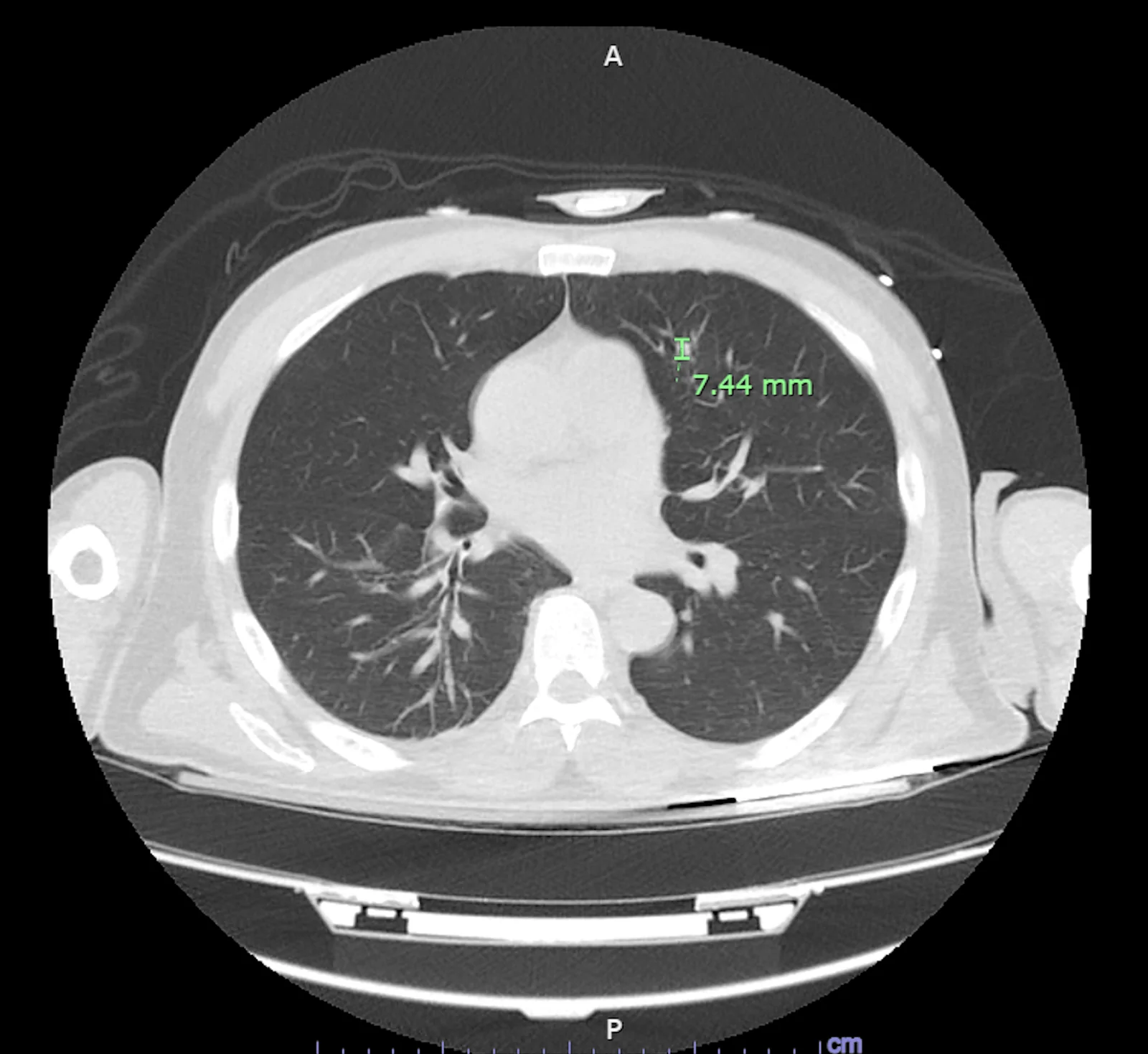
CT of the chest The CT shows an approximately 7-mm nodule versus nodular opacity in the left upper lobe (green marker), a finding that raised concern for an infectious versus malignant etiology.

**Figure 3:**
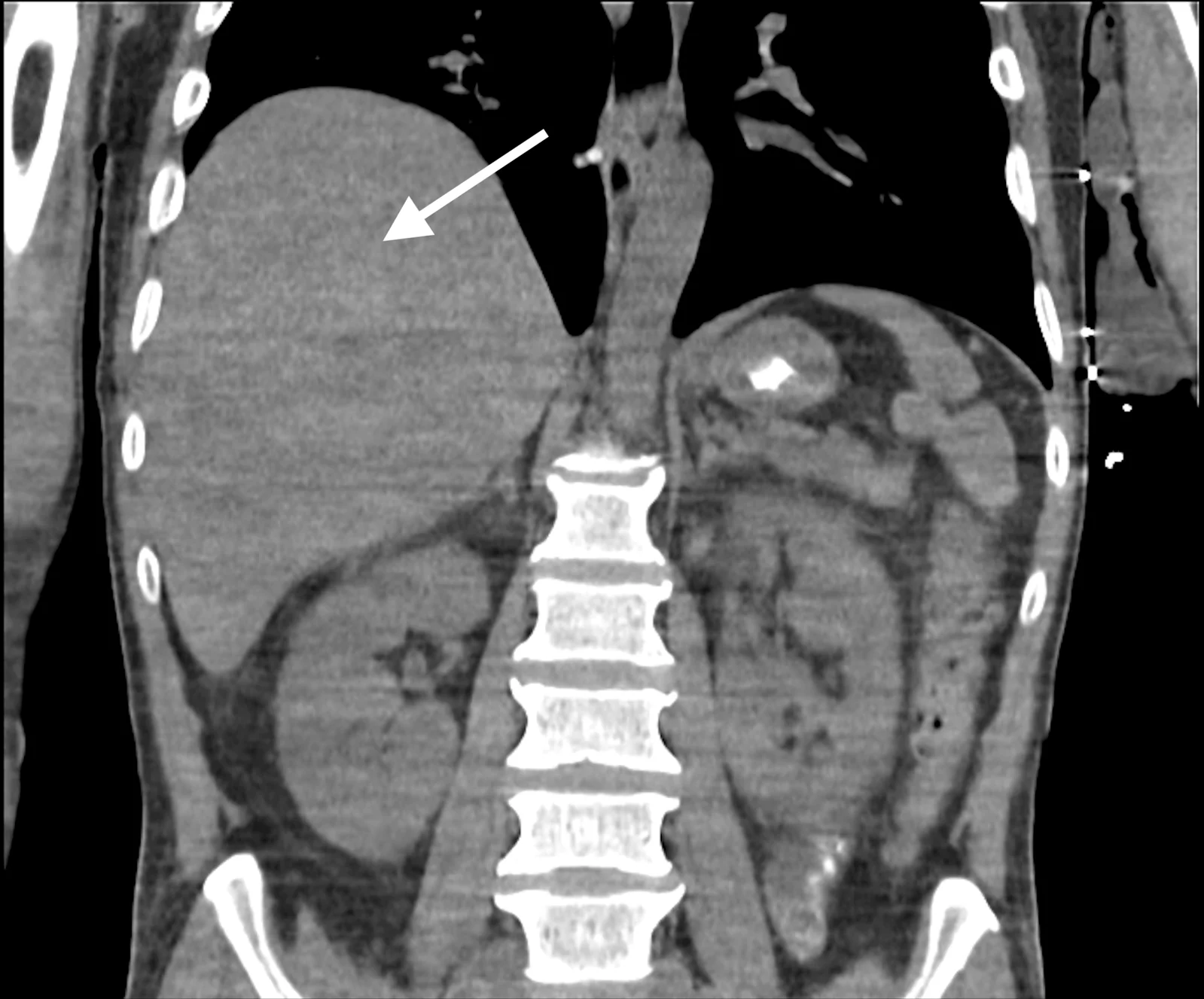
CT of the abdomen and pelvis Significant hepatomegaly (white arrow) without significant heterogeneity raised concern for hepatic pathology, and in the setting of an identified transaminitis, this raised additional concern for steatosis and hepatitis.

**Figure 4:**
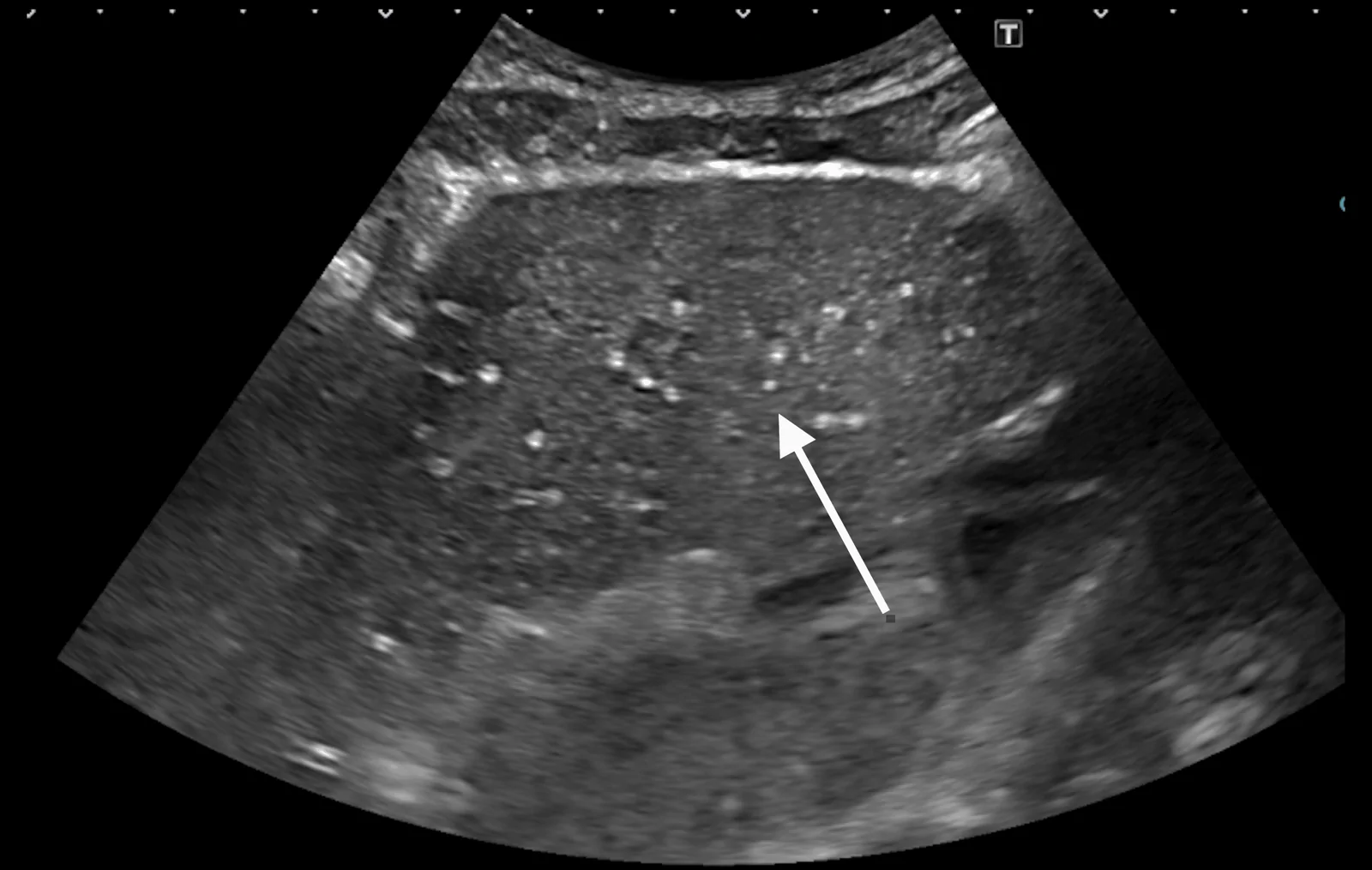
Right upper quadrant abdominal ultrasound The ultrasound shows hyperechoic hepatic parenchyma (white arrow), consistent with hepatic steatosis.

Given the hyperkalemia and acute renal failure, Nephrology was consulted, and urgent hemodialysis was performed. Initially, with his thrombocytopenia, there was concern for TTP, and Hematology was consulted; despite a lower risk PLASMIC score for TTP of 4 [16], urgent plasma exchange was recommended after the first round of dialysis. Hemolysis studies, DIC labs, and ADAMTS13 activity were sent beforehand. A blood smear showed occasionally large platelets. He was admitted to the medicine department with presumed sepsis from a gastrointestinal source, acute kidney injury, and thrombocytopenia.

The diagnostic course and clinical reasoning by hospital day are shown in Figure 5.

**Figure 5:**
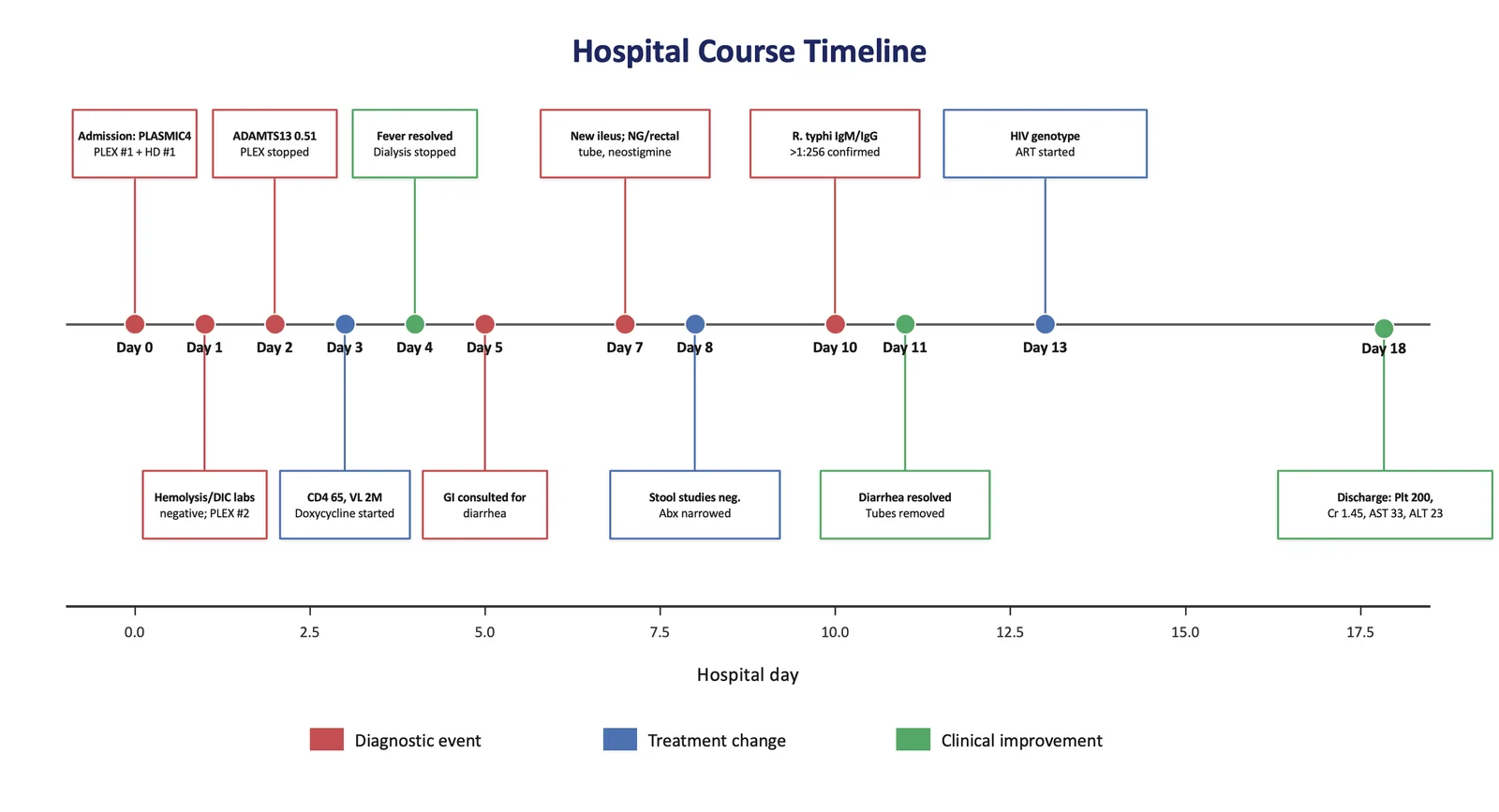
Admission timeline ADAMTS13, a disintegrin and metalloproteinase with a thrombospondin type 1 motif, member 13 (the enzyme whose deficiency causes TTP); ALT, alanine aminotransferase; ART, Antiretroviral Therapy; AST, Aspartate Aminotransferase; CD4, CD4+ T-cell count (marker of immune status in HIV); Cr, creatinine; DIC, disseminated intravascular coagulation; GI, gastroenterology (the consulting service); HD, hemodialysis; IgM/IgG, immunoglobulin M / immunoglobulin G (antibody classes); NG, nasogastric (tube); PLASMIC4, a clinical prediction score for TTP (thrombotic thrombocytopenic purpura) risk, based on 7 criteria (Platelet count, hemoLysis, Absence of active cancer, absence of Stem cell/solid organ transplant, MCV, INR, Creatinine; a score of 5 or higher suggests a moderate probability of severe ADAMTS13 deficiency); PLEX, plasma exchange (also called plasmapheresis); *R. typhi*, *Rickettsia typhi *(the causative organism of murine typhus); VL, viral load This timeline was generated on Microsoft PowerPoint.

Fever persisted into hospital day 1, prompting an infectious disease (ID) consultation while antimicrobials were continued. Laboratory testing showed no evidence of hemolysis or DIC, and a second round of plasma exchange and hemodialysis was performed while confirmatory ADAMTS13 results were still pending. These returned the following day, revealing a low but not critically deficient ADAMTS13 activity of 0.51 IU/mL (reference 0.68-1.63 IU/mL); with TTP now less likely, plasma exchange was discontinued. Antinuclear antibody was positive (1:40), but low specificity did not encourage further non-infectious workup at that time.

Despite this, fever continued into hospital day 3, and further workup uncovered the underlying driver of his presentation: a CD4 count of 65 cells/mm³ with an HIV viral load of two million copies/mL, confirming newly diagnosed AIDS. HLA-B*5701 testing was negative, and glucose-6-phosphate dehydrogenase activity was normal, clearing the way for antiretroviral therapy. With the constellation of fever, thrombocytopenia, transaminitis, acute kidney injury, altered mental status, and this newly recognized immunocompromised state, ID broadened the differential to include endemic rickettsial diseases, sending a Rickettsia antibody panel and RMSF IgM/IgG titers and starting empiric oral doxycycline 100 mg twice daily ahead of confirmatory results, given CDC-backed guidelines that treatment should be initiated based on clinical suspicion before laboratory confirmation. Ceftriaxone and metronidazole were continued pending stool culture, while intravenous vancomycin was stopped. Notably, his mentation cleared coincident with doxycycline initiation.

By hospital day 4, fever had resolved, and renal function had improved enough that dialysis was no longer needed. Non-bloody diarrhea, however, persisted, and by hospital day 5, Gastroenterology was consulted, and additional stool studies were sent. The clinical picture became more complicated on hospital day 7, when the patient developed tachycardia (heart rate in the 150s) and abdominal imaging revealed a new ileus; a nasogastric tube and a rectal tube were placed and intravenous neostigmine administered. Stool studies returned negative for *Cyclospora*, ova and parasite, *Giardia *antigen, *Campylobacter*, *Salmonella*, *Cryptosporidium*, Shiga-toxin *E. coli*, and *Isospora *on hospital day 8, allowing ceftriaxone and metronidazole to be discontinued.

The diagnostic picture crystallized on hospital day 10, when *R. typhi* ELISA IgM and IgG both returned positive at titers greater than 1:256, confirming murine typhus. RMSF ELISA IgM (1:128) and IgG (1:64) were also positive, but ID interpreted these, based on LA County Department of Health data showing at that time the endemic prevalence of *R. typhi* and not RMSF, as cross-reactive antibodies rather than a second rickettsial infection, and a 10-day doxycycline course was planned. Repeat testing for a fourfold rise was not performed. The following day, diarrhea resolved, and the nasogastric and rectal tubes and neostigmine were discontinued.

As renal function continued to improve, the focus shifted to HIV management: on hospital day 13, antiretroviral therapy with abacavir-dolutegravir-lamivudine was started at one tablet daily, and *Pneumocystis jirovecii* pneumonia prophylaxis was transitioned to trimethoprim-sulfamethoxazole double-strength, given three times a week (Monday, Wednesday, and Friday). By hospital day 18, having completed the 10-day doxycycline course, the patient's platelet count had recovered to 200 K/mm³, creatinine had improved to 1.45 mg/dL, and liver enzymes had largely normalized (Table 1); he was transferred to an acute rehabilitation facility.

**Table 1:** Laboratory and diagnostic findings Note: Values in bold fall outside the laboratory reference range. ADAMTS13, a disintegrin and metalloproteinase with a thrombospondin type 1 motif, member 13; ALT, alanine aminotransferase; AST, aspartate aminotransferase; CD4, cluster of differentiation 4; DIC, disseminated intravascular coagulation; Hb, hemoglobin; HIV, human immunodeficiency virus; HIV-1, human immunodeficiency virus type 1; HLA-B5701, human leukocyte antigen B57,01; IgG, immunoglobulin G; IgM, immunoglobulin M; INR, international normalized ratio; N/A, not applicable; PCR, polymerase chain reaction; R. typhi, Rickettsia typhi; RMSF, Rocky Mountain spotted fever

Test	Result	Reference range
Hematology/hemolysis (admission unless noted)		
White blood cell count	17.79 K/mm³	4.5–10 K/mm³
Hemoglobin	11.4 g/dL	13.5–16.5 g/dL
Platelets	44 K/mm³	160–360 K/mm³
Reticulocyte count	0.5%	0.6–2.4%
Lactate dehydrogenase	630 U/L	98–192 U/L
Haptoglobin	108 mg/dL	36–195 mg/dL
Direct antiglobulin test	Negative	N/A
Coagulation/DIC labs (admission)		
Prothrombin time	14.9 sec	11.9–14.6 sec
INR	1.17	0.87–1.14
Partial thromboplastin time	25.9 sec	22.9–33.1 sec
Fibrinogen	180 mg/dL	215–450 mg/dL
D-dimer	11.34 mcg/mL	<0.49 mcg/mL
ADAMTS13 activity (hospital day 2)	0.51 IU/mL	0.68–1.63 IU/mL
Chemistry/hepatic (admission)		
Creatinine	14.4 mg/dL	0.74–1.35 mg/dL
Potassium	6.5 mmol/L	3.5–5.0 mmol/L
AST	276 U/L	8–33 U/L
ALT	167 U/L	7–56 U/L
Total bilirubin	3.8 mg/dL	0.3–1.2 mg/dL
Direct bilirubin	2.2 mg/dL	0.1–0.5 mg/dL
HIV-related		
HIV-1 antibody	Positive	N/A
CD4 count (hospital day 3)	65 cells/mm³	N/A
HIV viral load	2,000,000 copies/mL	N/A
HLA-B*5701	Negative	N/A
Glucose-6-phosphate dehydrogenase	14.5 U/g Hb	7.0–20.5 U/g Hb
Rickettsial serology (hospital day 10)		
*R. typhi *IgM	Positive, titer >1:256	N/A
*R. typhi *IgG	Positive, titer >1:256	N/A
RMSF IgM	Positive, titer 1:128	N/A
RMSF IgG	Positive, titer 1:64	N/A
Discharge labs (hospital day 18)		
Platelets	200 K/mm³	160–360 K/mm³
Creatinine	1.45 mg/dL	0.74–1.35 mg/dL
Alkaline phosphatase	105 U/L	Lab-specific
AST	33 U/L	8–33 U/L
ALT	23 U/L	7–56 U/L

An extended infectious workup performed during hospitalization was otherwise unrevealing. Serologic and molecular testing was negative for *Cryptococcus*, *Coccidioides*, *Bartonella henselae* and *Bartonella quintana *DNA, *Mycobacterium tuberculosis *(QuantiFERON-TB Gold), *Cyclospora*, *Giardia *antigen, *Chlamydia trachomatis, *and *Neisseria gonorrhoeae *by polymerase chain reaction, syphilis, and hepatitis A immunoglobulin M and hepatitis B and C serologies. Cytomegalovirus immunoglobulin M was negative, with an immunoglobulin G greater than 10 U/mL consistent with prior exposure. Toxoplasma IgG was positive at 26.3, which is also consistent with prior exposure rather than acute infection.

At the infectious disease follow-up two months after discharge, the patient reported complete resolution of symptoms and adherence to antiretroviral therapy and prophylactic trimethoprim-sulfamethoxazole.

## Discussion

Murine typhus is caused by *R. typhi*, an intracellular gram-negative coccobacillus transmitted to humans by fleas, with rats and opossums serving as amplifying reservoirs rather than vectors [1]. The infection follows a distinct epidemiological pattern, disproportionately affecting urban, port, and coastal regions, including California [1]. It classically presents with a triad of fever, headache, and rash, alongside laboratory findings of elevated liver enzymes, lactate dehydrogenase, erythrocyte sedimentation rate, and hypoalbuminemia [7,8].

The clinical presentation of murine typhus often mimics other acute infectious causes, including scrub typhus, leptospirosis, dengue, and malaria, making a high index of suspicion essential [1]. Our patient's newly diagnosed AIDS, with a CD4 count of 65 cells/mm³, broadened this differential further and prompted an extensive infectious workup that excluded cryptococcosis, coccidioidomycosis, cytomegalovirus infection, and other opportunistic processes. This diagnostic ambiguity took a second, more consequential form: thrombocytopenia with a non-reassuring PLASMIC score for TTP [16], which lead to an initial working diagnosis of TTP and two rounds of empiric plasma exchange before ADAMTS13 activity returned negative, and plasma exchange was discontinued. This case illustrates the central diagnostic challenge: severe murine typhus can present with a laboratory and clinical picture that overlaps substantially with TTP, and distinguishing the two requires ADAMTS13 testing rather than clinical gestalt alone. Even when suspicion shifts toward a rickettsial cause, IgG antibody titers typically are not detectable until 7-10 days after symptom onset, with diagnostic confirmation resting on paired acute- and convalescent-phase titers [17]; therefore, empiric treatment must frequently precede serologic confirmation, as it did here.

Several recent case reports have described a similar pattern of murine typhus presenting with multi-organ involvement severe enough to prompt an alternative diagnosis. Wachs and Kuiper [18] reported a case of combined liver and renal failure requiring hemodialysis, with the correct diagnosis reached only after antimicrobial therapy had been empirically narrowed to doxycycline. Pervaiz et al. [19] described murine typhus presenting with DIC and acute psychosis, explicitly noting overlap with TTP-like presentations and recommending early neuroimaging to aid in diagnosis. This presentation, like our patient's altered mental status and intermittent confusion, underscores that neurologic involvement in murine typhus is not incidental; fulminant cases with status epilepticus and other severe neurologic sequelae have been reported [20]. A large recent cohort from Galveston, Texas (149 cases, 2019 through 2023) found that 28% of hospitalized patients required intensive care and that both deaths in the cohort involved multi-organ failure complicated by hemophagocytic lymphohistiocytosis [13], a reminder that severe murine typhus, independent of any comorbidity, carries a real risk of critical illness.

What distinguishes our case was the specific mechanism of diagnostic confusion: to our knowledge, this is among the more detailed accounts of murine typhus triggering a full TTP workup, including a validated pretest probability score and two rounds of plasma exchange, before ADAMTS13 testing redirected the diagnosis in a patient whose presentation ultimately spanned five organ systems: hematologic (thrombocytopenia), renal (acute kidney injury), hepatic (transaminitis), gastrointestinal (ileus), and neurologic (altered mental status and confusion). This combination, not the patient's HIV status per se, is what made this case diagnostically instructive and is the primary contribution we believe this report makes to the literature.

The pathogenesis of *R. typhi* centers on microvascular endothelial injury and vasculitis [1,5,6], resulting in increased vascular permeability, capillary leakage, and platelet consumption with microthrombi formation [6,21-23]. This microangiopathic process can manifest as kidney injury, thrombocytopenia, petechiae, or hepatic dysfunction [6,21,22]. In the renal system, this injury can result in prerenal azotemia, acute tubular necrosis, and, rarely, focal segmental glomerulosclerosis, as described in a previous murine typhus case report [5]. Within the gastrointestinal system, intestinal microvascular dysfunction and mucosal hypoperfusion can produce bowel wall edema, impaired motility, and ileus in severe infections [7,24,25], as occurred in our patient on hospital day 7, manifesting as tachycardia and requiring nasogastric decompression and neostigmine.

The severity of rickettsial infection depends, in part, on host factors; fatal outcomes have been associated with older or male adults and patients with diabetes, alcohol use disorder, or glucose-6-phosphate dehydrogenase deficiency [26]. Whether HIV/AIDS independently worsens murine typhus severity has not been established. A serosurvey of HIV-infected patients found *R. typhi *seroprevalence and clinical correlates similar to the general population [12], and a large case series characterizing complications of murine typhus explicitly excluded patients with untreated HIV infection from the analysis, alongside other confounding diagnoses [4], leaving this population understudied rather than demonstrating worse outcomes. The newly diagnosed AIDS broadened the initial differential diagnosis and likely contributed to the decision to pursue an aggressive infectious workup. However, the TTP-mimicking presentation and its resolution with doxycycline rather than plasma exchange are the features that make this case worth reporting, independent of the patient’s immunological status.

Doxycycline remains the cornerstone of therapy for murine typhus [27], and early empiric treatment, often initiated before confirmatory testing, as in the case reports above and in ours, is critical for reducing morbidity and mortality [1,13]. Within days of starting doxycycline, our patient improved: renal function normalized, the ileus resolved, thrombocytopenia resolved, mentation cleared, and no further intervention was required.

As a single case, this report is illustrative and hypothesis-generating rather than generalizable; it cannot establish causality between HIV/AIDS and murine typhus severity, and the absence of a convalescent-phase serology sample limits definitive confirmation of the diagnosis beyond a single elevated acute-phase titer.

## Conclusions

This case illustrates that severe murine typhus can closely mimic TMAs (TTP/HUS), presenting with thrombocytopenia and acute kidney injury. This presentation spanned five organ systems, underscoring that murine typhus can produce diffuse multisystem injury independent of any comorbidity.

Clinicians evaluating undifferentiated febrile illness with thrombocytopenia and multi-organ involvement should consider murine typhus in the differential diagnosis. The differential for fever with thrombocytopenia and multi-organ involvement is broad and extends well beyond TMA. Non-infectious mimics include hemolytic uremic syndrome and systemic autoimmune disease, while infectious mimics include leptospirosis, dengue, ehrlichiosis and anaplasmosis, acute HIV, acute viral hepatitis, Epstein-Barr virus and cytomegalovirus infection, secondary syphilis, and disseminated gonococcal infection. Many were pursued with directed serologic, molecular, and culture testing in this patient, and none of the overlapping clinical features separated *R. typhi* from its mimics at the bedside. In our patient, a newly diagnosed HIV infection broadened the initial infectious workup but was not established as the reason for his severe presentation. Prompt empirical doxycycline, initiated before serologic confirmation, is essential to prevent progression to critical illness.
